# Does suppression of oscillatory synchronisation mediate some of the therapeutic effects of DBS in patients with Parkinson's disease?

**DOI:** 10.3389/fnint.2012.00047

**Published:** 2012-07-10

**Authors:** Alexandre Eusebio, Hayriye Cagnan, Peter Brown

**Affiliations:** ^1^Department of Neurology and Movement Disorders, Assistance Publique - Hôpitaux de Marseille, Timone University HospitalMarseille, France; ^2^Institut de Neurosciences de la Timone – UMR 7289, Aix Marseille Université – CNRSMarseille, France; ^3^Department of Clinical Neurology, John Radcliffe HospitalOxford, UK

**Keywords:** Parkinson's disease, basal ganglia, deep brain stimulation, oscillations, neurophysiology

## Abstract

There is growing evidence for exaggerated oscillatory neuronal synchronisation in patients with Parkinson's disease (PD). In particular, oscillations at around 20 Hz, in the so-called beta frequency band, relate to the cardinal symptoms of bradykinesia and rigidity. Deep brain stimulation (DBS) of the subthalamic nucleus (STN) can significantly improve these motor impairments. Recent evidence has demonstrated reduction of beta oscillations concurrent with alleviation of PD motor symptoms, raising the possibility that suppression of aberrant activity may mediate the effects of DBS. Here we review the evidence supporting suppression of pathological oscillations during stimulation and discuss how this might underlie the efficacy of DBS. We also consider how beta activity may provide a feedback signal suitable for next generation closed-loop and intelligent stimulators.

## Introduction

Converging evidence from experimental and clinical studies indicates that deep brain stimulation (DBS) of the basal ganglia (BG) can be a very successful therapeutic approach in Parkinson's disease (PD). Accordingly, there has been a progressive shift from lesion techniques, which had been used for decades, to stimulation techniques, which have the major advantage of being—at least partly—reversible. Introduced in the early 1990's, DBS, particularly of the subthalamic nucleus (STN), is now a common therapeutic solution for advanced patients. However, the mechanisms underlying its remarkable efficacy in reducing parkinsonian symptoms remain largely unclear. Here, we will explore the hypothesis that DBS of the STN suppresses pathological synchronisation at low frequencies (13–35 Hz) and that this contributes to symptomatic benefit. To this end we will begin with a critical discussion of the evidence that such oscillatory synchronisation underlies motor impairment in PD and briefly discuss the efficacy of current high-frequency DBS regimes, before considering the evidence that DBS does suppress oscillatory synchronisation.

## Excessive oscillations in the beta band are a neurophysiological signature of patients with parkinson's disease

There is extensive evidence indicating that abnormally synchronised activity patterns in the beta band (13–35 Hz) are a hallmark of untreated PD. These activities have principally been found in recordings from the STN and globus pallidus interna (GPi) of PD patients withdrawn from their medication. Activities synchronised in this band have been captured in single-unit recordings and cross-correlograms of neuron pairs (Levy et al., 2002a,b; Amirnovin et al., 2004; Kuhn et al., 2005). They have also been recorded in the local field potentials (LFPs) from striatum (Sochurkova and Rektor, 2003), STN (Brown et al., 2001; Priori et al., 2002, 2004; Williams et al., 2002, 2003, 2005; Kuhn et al., 2004; Bronte-Stewart et al., 2009), and GPi (Brown et al., 2001; Priori et al., 2002; Silberstein et al., 2003). Oscillations recorded from the STN and GPi are ipsilaterally coherent in the beta band (Brown et al., 2001; Cassidy et al., 2002; Foffani et al., 2005) and are coherent with electroencephalographic and magnetoencephalographic activities recorded over motor cortex in the same frequency band (Marsden et al., 2001; Williams et al., 2002; Lalo et al., 2008; Hirschmann et al., 2011; Litvak et al., 2011). In addition, it has recently been shown that beta oscillations are coherent bilaterally between the two STNs suggesting existence of a strong functional connection between these nuclei, although the exact anatomy of this connection remains to be established (de Solages et al., 2010).

The beta frequency band is broad and there may be functionally important subdivisions within it (Priori et al., 2004; Marceglia et al., 2006, 2007; Lopez-Azcarate et al., 2010). In particular, LFP power in the lower (<20 Hz) beta band is more dramatically suppressed by levodopa and apomorphine than that in the upper (>20 Hz) beta band (Priori et al., 2004; Marceglia et al., 2006). Such distinctions would be consistent with the existence of several oscillating circuits, each with its own resonance frequency. There is evidence that activity in the STN is preferentially coupled with mesial cortical areas in the upper beta frequency band, while correlated with sensorimotor cortex in the lower beta band (Fogelson et al., 2006). These findings are mirrored in the distribution of cortical beta activity in healthy subjects (Pfurtscheller et al., 2003). Accordingly, different BG-cortical loops may be effectively tuned to activities in different frequency bands.

What is the evidence that oscillatory activity in the beta frequency band may contribute to motor impairment in patients with PD? There are several reports on levodopa-induced suppressions of beta LFP power and the incidence of neurons oscillating over this frequency range in the STN correlates with treatment induced improvement in bradykinesia and rigidity, but not tremor (Kuhn et al., 2006, 2009; Weinberger et al., 2006; Ray et al., 2008). Less clear is whether oscillatory activity in the beta band off medication state correlates with motor impairment in the off medication state. Reports vary with respect to whether they found (Chen et al., 2010; Lopez-Azcarate et al., 2010; Pogosyan et al., 2010; Ozkurt et al., 2011) or did not find such a correlation (Kuhn et al., 2006, 2009; Weinberger et al., 2006; Ray et al., 2008). This variability may relate to the absence of normalisation in some studies to offset the effects of microsubthalamotomy and targeting variance between sides and patients (Chen et al., 2010). Some investigations have also reported a correlation between level of activity in the beta band off medication and levodopa-induced improvement in bradykinesia and rigidity (Weinberger et al., 2006; Zaidel et al., 2010).

Additional correlative evidence comes from the observation that the distribution of beta oscillations within the STN is concentrated in its dorsolateral motor territories (Kuhn et al., 2005; Alonso-Frech et al., 2006; Weinberger et al., 2006, 2009) and a recent study has shown that the therapeutic outcome of DBS in PD is predicted by the extent of the oscillatory activity in this region (Zaidel et al., 2010). It has also been shown that DBS is more efficient in reducing bradykinesia and rigidity when applied exactly where beta oscillations are maximal (Yoshida et al., 2010).

However, the above correlative evidence does not prove that beta oscillations are mechanistically important to movement impairment. Proof-of-principle that excessive beta synchrony is causal would require the demonstration of motor impairment induced by STN stimulation at beta band frequencies. STN DBS at 10 Hz increases bradykinesia as assessed by section III of the United Parkinson's Disease Rating Scale (UPDRS) (Timmermann et al., 2004). Other studies, which utilise simple finger tapping tasks, show relative deteriorations in movement performance during stimulation in the 5–10 and 20–25 Hz frequency bands (Fogelson et al., 2005), particularly when those patients with baseline performances within the normal range are considered (Chen et al., 2007; Eusebio et al., 2008). Baseline performance level is correlated with the extent of motor impairment possibly because subjects with better performance are less likely to be already swamped by excessive synchrony at low frequencies. It should also be noted that deleterious effects of stimulation at low frequency are relatively frequency selective, as 50 Hz stimulation does not impair movement (Chen et al., 2007).

Adverse effects of low frequency stimulation have been modest in the above investigations, but a recent study assessing the rate of force generation in maximal hand grips reported impairments of about 15%, rising to 22% when only those patients with better performance at baseline were considered (Chen et al., 2011). Even this falls short of the 50% or so improvement following levodopa administration or high-frequency stimulation in the same group of patients. One possible reason for the differences in the extent of motor performance changes during high-frequency and low frequency stimulation could be insufficient temporal patterning of low frequency stimulation; application of high-frequency bursts of pulses at ~20 Hz might be necessary to effectively amplify neural synchrony and lead to more severe motor impairments (Brown, 2007). Nonetheless, these data do leave open the possibility that, even if DBS suppresses low frequency oscillations, its full effect may not be solely mediated through this.

## STN DBS in parkinson's disease

The modern history of DBS of BG nuclei essentially starts with observations made by Benabid and Pollak in Grenoble, although earlier observations had been made (Hariz et al., 2010). The clinical effect of stimulation was initially incidentally observed during a thalamotomy procedure where stimulation was applied for localisation purposes. During surgery, the operator noticed that stimulation when applied at frequencies greater than 100 Hz produced a reversible effect on the tremor (Benabid et al., 1987). The efficacy of chronic stimulation applied at the ventral intermediate nucleus of the thalamus in producing a sustained tremor reduction was later confirmed in a larger group of patients (Benabid et al., 1991). In the early 1990's, experimental data in the parkinsonian non-human primate led to the suggestion that lesioning or stimulating the STN at high-frequency could relieve parkinsonian symptoms (Bergman et al., 1990; Benazzouz et al., 1993). It did not take long before this effect was confirmed in patients (Benabid et al., 1994; Limousin et al., 1995). Since then, several thousands of patients have been operated upon and numerous studies have confirmed the efficacy and the safety of the DBS in PD with some target-dependant specificities (The deep-brain stimulation for Parkinson's disease study group, 2001; Rodriguez-Oroz et al., 2005; Deuschl et al., 2006).

In particular, the long-term efficacy of STN DBS in reducing both motor and some non-motor symptoms of PD has been extensively documented worldwide in large patient series (The deep-brain stimulation for Parkinson's disease study group, 2001; Krack et al., 2003; Rodriguez-Oroz et al., 2005; Deuschl et al., 2006; Witjas et al., 2007; Fasano et al., 2010; Follett et al., 2010) as well as in a meta-analysis of over 900 patients (Kleiner-Fisman et al., 2006). The average long-term improvement in motor symptoms assessed using the UPDRS III scale is about 50%—with a higher efficacy on tremor and rigidity than on bradykinesia (Krack et al., 2003). The reduction in levodopa dose is also about 50% (Benabid et al., 2009). Levodopa-induced dyskinesias are reduced by 60% after surgery (Krack et al., 2003) although the mechanisms by which this occurs—levodopa reduction or a direct effect of DBS—remains debated. Quality of life improves shortly after STN DBS (Deuschl et al., 2006) but, in contrast to sustained motor improvements, this effect seems to disappear with time (Volkmann et al., 2009). Both STNs are generally implanted with electrodes since PD symptoms at this stage of the disease are bilateral. However, there is some evidence to suggest that unilateral STN DBS may have bilateral effects (Tabbal et al., 2008; Walker et al., 2009) even though these may not last (Kim et al., 2009).

## Evidence for suppression of beta by high-frequency STN DBS

Although DBS is remarkably effective in treating movement disorders such as PD, dystonia and essential tremor, the mechanism underlying this efficacy remain essentially unexplained. In PD, DBS mimics the effects of lesioning and was therefore initially considered to inhibit STN or GPi output in keeping with the classic pathophysiological model of the disease (Albin et al., 1989; DeLong, 1990). Consistent with this, *in vitro* recordings in slice preparations from parkinsonian rats showed a frequency-dependent decrease in neuronal activity during and after STN stimulation (Beurrier et al., 2001). This effect was also seen *in vivo* in the parkinsonian primate (Meissner et al., 2005). Locally decreased neuronal activity was also seen during STN and GPi stimulation of PD patients (Dostrovsky et al., 2000; Welter et al., 2004). In addition, at the cellular and synaptic level, there is evidence to suggest that high-frequency stimulation of the STN reverses some of the abnormalities observed in PD and in particular glutamatergic hyperactivity consistent with a blockade of STN activity (Salin et al., 2002; Gubellini et al., 2006). The suppressive role of STN DBS on neuronal activity is, however, challenged by the observation that, activity in the SNr, which is a major target nucleus of the STN, is increased during high-frequency STN stimulation (Maurice et al., 2003). Additionally, recordings obtained from parkinsonian primates showed a marked increase in GPi activity during STN stimulation at 130 Hz (Hashimoto et al., 2003). It was more recently shown that STN DBS does not in fact silence STN neurons in PD patients but rather changes their firing pattern (Carlson et al., 2010). These apparently contrasting effects can be reconciled by the observation that DBS is capable of inhibiting the spontaneous firing of STN and simultaneously generate a new pattern of activity (Garcia et al., 2003). This is consistent with the observation that DBS does not have the same impact on the neuronal soma and axon, suggesting that STN DBS could uncouple somatic and axonal activity (Holsheimer et al., 2000; McIntyre et al., 2004).

Nevertheless, these observations regarding the effects of high-frequency DBS on neuronal activity (Garcia et al., 2005; Hammond et al., 2008) have failed to provide a satisfactory explanation for the paradoxical effects of DBS on motor function highlighted over a decade ago (Marsden and Obeso, 1994). One hypothesis is that PD leads to a pattern of BG activity that disrupts local and distant function and that DBS acts to suppress this “noisy signal” (Brown and Eusebio, 2008). In PD, the noisy signal could be excessive beta synchronisation and one tentative hypothesis would therefore be that STN DBS suppresses beta oscillations in the STN as well as in its projection targets. Several computational studies corroborate this hypothesis and suggest that the efficacy of STN DBS could depend on regularization of activity patterns and suppression of synchronized oscillatory activity patterns observed in down-stream nuclei (Rubin and Terman, 2004; Guo et al., 2008; Cagnan et al., 2009; Dorval et al., 2010; Hahn and McIntyre, 2010) and that the stimulation amplitude and frequency dependency of DBS efficacy could stem from whether stimulation is able to effectively suppress and/or regularize the pathological oscillations observed in the BG (Cagnan et al., 2009; Hahn and McIntyre, 2010). In a recent study, Dorval et al. highlighted that replacing the state of the art regular 130 Hz stimulation with an irregular stimulation pattern (with a mean frequency of 130 Hz and SD of 78 Hz), is not able to reduce bradykinesia in patients with PD and concurrently suggested, using a computational model that this could stem from the ineffectiveness of irregular high-frequency stimulation in regularizing BG activity (Dorval et al., 2010).

The evidence that DBS may modulate beta activity in patients with PD is mixed and mostly indirect. The latter is because simultaneous recordings of STN LFPs during DBS were, until recently, obviated by stimulation-induced electrical artifacts which are several orders of magnitude larger than the spontaneous fluctuations of the LFP (Rossi et al., 2007). So investigators have recorded at projection sites of the STN, where stimulation artifact is less of a problem. STN DBS-induced improvement in bradykinesia and rigidity (but not tremor) correlates with the degree of suppression of synchronisation in the beta band at the cortex (Silberstein et al., 2005) and STN DBS suppresses beta LFP activity in the pallidum (Brown et al., 2004). Another way round the problem of stimulation artifact has been to record the immediate after effects of STN DBS in those patients with a delayed return of bradykinesia and rigidity upon cessation of DBS. Here results have been mixed. Several studies reported a suppression of beta activity while therapeutic effects last (Wingeier et al., 2006; Kuhn et al., 2008; Bronte-Stewart et al., 2009), with the suppression of beta activity following cessation of DBS correlating with the degree of residual motor improvement (Kuhn et al., 2008). However, suppression of beta activity was not replicated in a further study (Foffani et al., 2006). The authors of the latter study have since developed a specially designed amplifier which enables LFP recordings simultaneously with stimulation of the same site (Rossi et al., 2007). They have gone on to record from the STN directly during DBS and again failed to show significant suppression of LFP power in the beta band (Rossi et al., 2008). Although this study was a technological feat, not all recordings had peaks in the beta band prior to DBS so that power suppression may have been difficult to detect in these cases, a problem compounded by the recording of some patients on medication. Indeed the same groups have since published a study showing that high-frequency STN DBS does suppress simultaneously recorded beta oscillations, albeit to a lesser extent than with treatment with levodopa (Giannicola et al., 2010).

**Figure 1:**
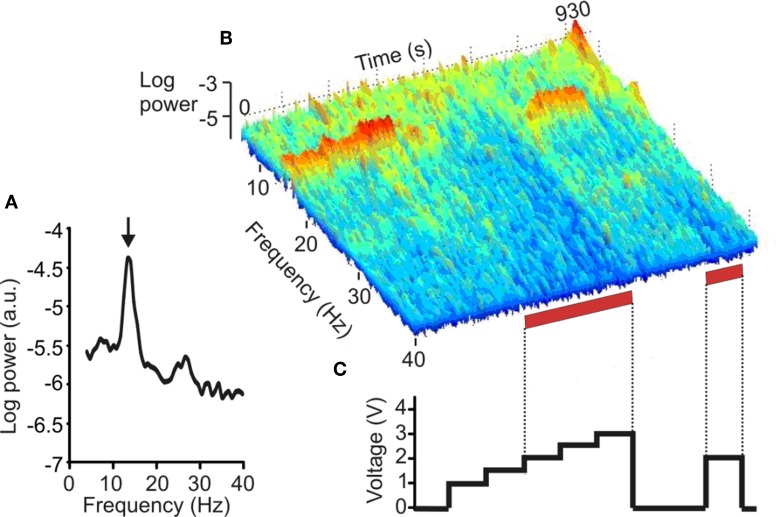
**Effect of DBS of the STN on the LFP. (A)** Power autospectrum of LFP recorded without stimulation. There is a large peak arrowed at 14 Hz. **(B)** Frequency-time log power spectrum of LFP (contact pair 02). Red bars along the time axis denote periods of DBS at 2.0–3.0 V, which induced motor improvement but also dyskinesias of the contralateral foot. Note the suppression of the 14 Hz peak with stimulation = 2.0 V, and the delayed return of the 14 Hz activity after stimulation is terminated. **(C)** Timing and voltage of DBS applied at contact 1. (Adapted with permission from Eusebio et al., 2011).

A further, more recent, investigation used a similar approach, but restricted study to the effects of STN DBS in a large sample of PD patients with evidence of pathological synchrony in the subthalamic region at baseline, prior to stimulation. DBS progressively suppressed peaks in LFP activity at frequencies between 11 and 30 Hz as stimulation voltage was increased beyond a threshold of 1.5 V. The latter corresponded to the median threshold for clinical efficacy of high-frequency STN DBS in these patients (Figure 1). The study provides strong evidence that DBS can suppress pathological activity at 11–30 Hz in the vicinity of stimulation in patients with PD and that this suppression occurs at stimulation voltages that are clinically effective (Eusebio et al., 2011). The suppression of pathological beta oscillations may therefore provide a common mechanism of action for both levodopa and STN DBS.

## Limitations of the theory that high-frequency STN DBS suppresses local beta activity

It cannot, however, be assumed that STN DBS-induced beta suppression is limited to the STN region, or that this is the sole consequence of stimulation. It is possible that DBS influences neuronal activity both locally at the site of stimulation (in and around STN), and also over other functionally connected elements of the cortex—BG network (Hashimoto et al., 2003; Brown et al., 2004; Dorval et al., 2008; Hammond et al., 2008; Kuhn et al., 2008; Montgomery and Gale, 2008). Such propagation may be both orthodromic and antidromic. For example, the bilateral efficacy of unilateral STN DBS could potentially be partly explained by the coupling of beta oscillations across the two STN (de Solages et al., 2010). The propagated effect of STN DBS on the BG—thalamocortical network has been extensively studied in several computational studies (Rubin and Terman, 2004; Guo et al., 2008; Cagnan et al., 2009; Pirini et al., 2009; Dorval et al., 2010; Hahn and McIntyre, 2010). These studies have been founded on the hypothesis that thalamus plays a key role in PD pathophysiology by relaying aberrant BG oscillations to cortex, forming a closed circuit via the hyper-direct pathway from the cortex to the STN resonating at beta band frequencies. It has been suggested that high-frequency stimulation of the STN is effective since it restores thalamic relay fidelity (Rubin and Terman, 2004; Guo et al., 2008; Dorval et al., 2010) and disrupts relay of pathological oscillations to cortex via thalamus (Cagnan et al., 2009). It has also been highlighted that activity patterns rather than rate are vital in determining modulatory effects of BG output on thalamus and the overall efficacy of DBS (Rubin and Terman, 2004; Guo et al., 2008; Cagnan et al., 2009; Pirini et al., 2009; Dorval et al., 2010).

The mechanisms by which STN DBS might induce beta suppression are also unclear. There is evidence to suggest that dopamine attenuates beta oscillations through the damping of resonance phenomena in local circuits (Eusebio et al., 2009). This may occur through the alteration of voltage-dependent conductances (Nicola et al., 2000) that act to dampen oscillations (Gutfreund et al., 1995). It seems plausible therefore that STN DBS might similarly promote damping of circuit resonance in the beta band. There is evidence both from *in vitro* recordings and computational studies to suggest that high-frequency DBS may modulate local currents and, in particular, potassium and sodium conductances (Beurrier et al., 2001; Shin and Carlen, 2008; Cagnan et al., 2009).

Although STN DBS seems to suppress pathological beta activity, this need not imply that STN DBS-induced motor improvement is exclusively due to beta suppression. Certainly a minority of patients do not have spectral peaks in the beta band during the post-operative period (Eusebio et al., 2011). Moreover, there is increasing evidence that beta activity is not directly linked to parkinsonian tremor (Silberstein et al., 2003; Amirnovin et al., 2004; Kuhn et al., 2006, 2008, 2009; Weinberger et al., 2006; Ray et al., 2008), although one early study found a association between rest tremor and 15–30 Hz oscillations (Levy et al., 2000). This presents a problem, as STN DBS is very effective in suppressing Parkinsonian tremor. Perhaps, elevation of beta is a necessary pre-requisite for tremor, but not, by itself, sufficient to cause it. Alternatively, tremor may relate to oscillatory activities in the BG at other frequencies that are also simultaneously suppressed by DBS. Two candidate frequencies have been suggested, and need not be mutually exclusive. First, there are those oscillations at the frequency of rest tremor, 4–6 Hz (Weinberger et al., 2009), and its harmonic, 8–12 Hz (Rivlin-Etzion et al., 2006). Second, tremor periods are associated with an elevation in low gamma (35–55 Hz) power in the LFP (Weinberger et al., 2009). Figure 1 suggests that the suppression of LFP activities by STN DBS at therapeutic voltages may be relatively indiscriminate and extend to both sub-beta band and low gamma activities, although the amplifier design precludes assessment of frequencies over 40 Hz.

The potential local suppression of low gamma activity by STN DBS should be distinguished from its potential effects at even higher frequencies. Thus, it has been suggested that some of the effects of DBS may relate to the driving of neurons and axons near the point of stimulation (Garcia et al., 2005; Hammond et al., 2008; Montgomery and Gale, 2008). STN DBS may drive subthalamic activity at stimulation frequency (Meissner et al., 2006), with this activity then being propagated to other sites (Hashimoto et al., 2003; Brown et al., 2004). This effect might mimic the increase in synchrony at high gamma (>55 Hz) frequencies that follows treatment with levodopa (Brown et al., 2001; Foffani et al., 2003), although the evidence that high-frequency oscillations are functionally relevant in restoring normal motor function in PD patients is not so far compelling.

## Implications for the future of DBS therapy

Although current fixed stimulation DBS regimes afford good control of motor symptoms in PD, they are by no means perfect. In particular, some side-effects may relate to the indiscriminate suppression or over-riding of residual physiological functioning in BG-cortical circuits (Chen et al., 2006; Ray et al., 2009). A recent study demonstrated that DBS-induced side-effects can be minimized by using field steering and constraining stimulated regions (Frankemolle et al., 2010). An alternate approach to improve DBS is to introduce closed-loop therapy, which involves continual monitoring of clinical state or surrogate neuronal activity, so that stimulation need only be delivered when necessary. Here the idea is that periods of non-stimulation might allow physiological functioning with limitation of side-effects, less accommodation to the effects of stimulation and savings in battery power. Indeed, there is evidence that both the neuronal activity in the BG and the motor symptoms are highly variable with time (Brown, 2003; Hammond et al., 2007; Raz et al., 2000), potentially allowing for intermittent therapy, especially as most patients also still receive dopaminergic medication. Furthermore, adjusting the stimulation parameters frequently has been shown to better improve symptom control (Moro et al., 2006). Recently, the group of Bergman constructed a closed-loop stimulator and compared its efficacy in reducing motor impairment in parkinsonian primates to that of continuous high-frequency stimulation. The device used the activity of cortical neurons to determine when to deliver short trains of high-frequency simulation to the GPi. They found that the closed-loop strategy was superior to standard DBS in both alleviating the symptoms and suppressing the pathological oscillatory activity (Rosin et al., 2011). As well as providing further support for the pathological role of oscillations in PD these important results pave the way for future studies in patients. Nevertheless, the approach taken in this proof-of-principle study may not be the most suitable for clinical translation. This is because of difficulties in maintaining recordings of the activities of single neurons over time and the necessity for two surgical targets (cortical and pallidal). The strong correlation between beta activity and bradykinesia-rigidity suggests that this might provide an alternative biomarker, evident in the LFP and therefore more robust over time, and able to be detected from the very site of stimulation (Stanslaski et al., 2009). Importantly, and as discussed here, this beta activity is likely to be suppressed by successful DBS, a critical feature of any signal to be used for feedback control of DBS.

The advantage of the above approach is that it relies only on a strong correlation between beta activity and motor impairment at any given time. However, the growing evidence that exaggerated beta synchrony may be mechanistically linked to motor impairment in PD suggests another and more sophisticated approach. Here, the temporal patterning of stimulation is organised so that it specifically targets the putative pathological activity. There are several approaches that can be taken (Tukhlina et al., 2007; Hauptmann and Tass, 2010; Guo and Rubin, 2011), but perhaps the most intuitive is phase cancellation as practised in noise reducing headphones. The advantage is that this would target pathological activity but leave physiological processing relatively untouched, thereby limiting side-effects.

In conclusion, we have reviewed evidence that high-frequency STN DBS suppresses excessive beta synchrony in PD and that this is—at least in part—responsible for the improvement in motor symptoms. Several issues remain unresolved and in particular the mechanisms by which STN DBS suppresses beta oscillations and the possibility that other consequences of high-frequency STN DBS—unrelated to beta suppression—may contribute to symptomatic improvement.

### Conflict of interest statement

Peter Brown is a consultant to Medtronic Ltd. Hayriye Cagnan and Alexandre Eusebio have no commercial or financial relationships that could be construed as a potential conflict of interest.
